# The effect of bispectral index-controlled sedation on postoperative cognitive health in elderly patients with spinal anesthesia undergoing elective total knee arthroplasty: randomized, blinded study

**DOI:** 10.1186/s44158-026-00437-0

**Published:** 2026-07-23

**Authors:** Inna Jaremko, Gabrielė Milkintaitė, Arūnas Gelmanas, Alfredas Smailys, Tadas Lenkutis, Andrius Macas

**Affiliations:** 1 Department of Anesthesiology, Faculty of Medicine, Medical Academy, Lithuanian University of Health Sciences, Kaunas, Lithuania; 2 Department of Orthopaedics and Traumatology, Faculty of Medicine, Medical Academy, Lithuanian University of Health Sciences, Kaunas, Lithuania

**Keywords:** BIS-guided sedation, Cognitive function, Elective knee arthroplasty, Elderly, Spinal anesthesia, Preoperative anxiety

## Abstract

**Background:**

Postoperative cognitive dysfunction (POCD) commonly affects elderly patients, but the impact of BIS-guided sedation during elective orthopedic surgery under spinal anesthesia remains unclear. The aim was to compare BIS-controlled versus standard clinically guided sedation on POCD in elderly patients undergoing elective knee arthroplasty.

**Methods:**

This was a prospective, patient- and outcome assessor-blinded, randomized controlled clinical study. Participants were randomized into two groups: BIS-targeted sedation (BIS group > 70) and standard clinical sedation (non-BIS group). All patients received the same anesthetic and analgesic. The primary outcome was the change in cognitive function over time. Cognitive function was assessed four times using the Addenbrooke’s Cognitive Examination–Revised (ACE-R) questionnaire. Higher scores indicate better cognitive performance. The emotional status was evaluated two times using the Hospital Anxiety and Depression Scale (HADS)*.*

**Results:**

Ninety-two patients (46 per group) were included in the final analysis. No statistically significant differences in total ACE-R score were found between the two groups at any time point (*p* > 0.05). Within the BIS group, total ACE-R scores were significantly higher on postoperative day (POD) 90 than preoperatively (PO) (87 (65–96) vs. 92 (70–98), *p* = 0.007). In the non-BIS group, total ACE-R scores increased significantly at several time points: from PO to 30 POD (87 (48–96) vs. 90.5 (40–98), *p* = 0.007), from PO to 90 POD (87 (48–96) vs. (91 (40–99), *p* < 0.001), and from 2 to 30 POD (88 (45–98) vs. 90.5 (40–98), *p* = 0.005). Significant decline in the number of patients experiencing probable anxiety at 30 POD compared with PO in both groups (BIS 28.3% to 2.2%, *p* = 0.004; non-BIS 32.6% to 8.7%, *p* = 0.015).

**Conclusions:**

This study did not demonstrate superiority of BIS-controlled sedation compared to clinically guided sedation with regard to cognitive outcomes. Further studies are needed.

**Trial registration:**

ClinicalTrials.gov (NCT06241859, January 28, 2024)

## Background

Postoperative cognitive dysfunction (POCD) is a common and unpleasant postoperative complication in elderly patients. The incidence of POCD varies widely depending on population, surgery type and urgency, type of anesthesia, assessment methods, and timing. Epidemiological evidence indicates that POCD affects 40% of older adults at the time of hospital discharge [1]. According to a recent systematic review [2], the prevalence of POCD in older noncardiac surgical populations is about 23% at 7 days, 16% at 1 month, and around 10% at 3 months**.** The pathophysiology of POCD is multifactorial, including age-related cognitive deterioration, perioperative and anesthetic factors, systemic and central nervous system inflammation, as well as genetic and environmental influences [3].

Total knee arthroplasty (TKA) is among the most frequently performed orthopedic procedures in older adults and is often performed under spinal anesthesia (SA) with intraoperative sedation [4]. Despite the benefits of regional anesthesia, elderly patients remain at risk of postoperative cognitive dysfunction (POCD) [1, 5]. Early POCD after TKA has been reported in up to 72% of patients, with persistent impairment in about 30% at 6 months [6]. A narrative review of elective hip and knee arthroplasty found a median incidence of early POCD of approximately 19.3% at 1 week and 10% at 3 months postoperatively [7]. Preservation of cognitive function in this population is therefore an important clinical goal, as POCD has been associated with complications and prolonged hospital stay [8, 9], delayed rehabilitation [9, 10], and reduced quality of life.

Sedation during regional anesthesia is routinely administered to improve patient comfort. Propofol-based sedation, although widely used for its rapid onset, short duration, and low cost, may contribute to cognitive postoperative cognitive dysfunction (POCD) if not carefully titrated [11, 12]. Excessive depth of sedation has been identified as a potentially modifiable risk factor for POCD, particularly in older patients [13, 14]. The bispectral index (BIS) provides an objective, electroencephalography-based measure of hypnotic depth and has been shown to reduce anesthetic exposure and the incidence of excessively deep sedation [15, 16]. However, the impact of BIS-guided sedation on postoperative cognitive outcomes in elderly patients undergoing elective orthopedic surgery under spinal anesthesia remains unclear. We hypothesized that BIS-targeted moderate-to-light sedation would have a significant positive effect on postoperative cognitive outcomes over time compared with standard clinical sedation.

This prospective, patient- and outcome assessor-blinded, randomized controlled study aimed to compare the impact of BIS-controlled sedation with standard sedation, guided by clinical signs, on postoperative cognitive function in patients undergoing elective knee arthroplasty under spinal anesthesia.

## Methods

This was a prospective, patient- and outcome assessor-blinded, randomized controlled clinical study conducted at the Department of Anesthesiology of the Lithuanian University of Health Sciences Hospital Kauno Klinikos (LSMUL KK). The study was approved by the Regional Bioethics Committee of Lithuania (Protocol No. BE-2–53, on May 11, 2021) and was conducted in accordance with the principles of the Declaration of Helsinki. In addition, the study protocol was registered at ClinicalTrials.gov (NCT06241859) on January 28, 2024.

### Study participants

A research coordinator approached eligible patients before their scheduled surgery. Inclusion criteria: (1) elective knee replacement surgery under SA with sedation; (2) age ≥ 65 years; (3) American Society of Anesthesiologists (ASA) physical status I–III; and (4) willingness to participate in the study. Exclusion criteria (1) refusal of or contraindication to undergo SA with sedation (2) previously diagnosed cognitive disorders; (3) at preoperative evaluation mini-ACE < 22 points; (4) current use of benzodiazepines (BZDs) or other psychotropic medications; (5) hearing, vision or speech impairments that could interfere with completion of study questionnaires; (6) inability to read, write or understand Lithuanian; (7) refusal to participate in the study. All participants signed a written informed consent.

Before the surgery, all participants were randomized with allocation concealment into two groups: one group receiving BIS-targeted sedation (BIS > 70) and the other undergoing standard sedation based on clinical signs, using the Richmond Agitation-Sedation Scale (RASS) within the range of moderate-to-light sedation (BIS group and non-BIS group, respectively). Participants were randomized using a sealed opaque envelope method (simple randomization). The envelopes were prepared by an investigator using a 1:1 randomization ratio and were opened only after patient enrollment. The anesthesiologist responsible for intraoperative data collection was informed of the sedation method administered; however, both the patients and the independent outcomes assessor remained blinded.

### Perioperative management

During the perioperative period, all patients in both groups received the same anesthetic and analgesic regimen according to our institutional clinical protocol. Intraoperative monitoring, including electrocardiography, pulse oximetry, noninvasive blood pressure, and BIS monitoring, was initiated. Premedication with intravenous midazolam 2.5 mg and dexamethasone 8 mg was given to all patients, and a slow crystalloid infusion with 1 g of tranexamic acid and 10 mg of ketamine was started 30 min before surgery. SA was performed at the L3–L4 intervertebral space using 15 mg of levobupivacaine with the patient in the lateral decubitus position. Subsequently, a peripheral nerve block (the apex of the femoral triangle and the distal adductor canal) for postoperative analgesia was performed under ultrasound guidance. During the surgery, patients received intravenous propofol sedation. The initial propofol dose was 0.5 mg/kg, followed by continuous infusion according to group allocation: in the non-BIS group, sedation depth was adjusted to maintain a RASS score between − 3 and − 2, whereas in the BIS group, sedation was titrated to maintain a BIS score > 70. In the case of hypotension (systolic blood pressure < 90 mmHg), 5 mg of ephedrine was administered.

All arthroplasties were performed with cemented prostheses through a medial parapatellar approach, without patellar resurfacing. For pain management, all patients received local infiltration analgesia comprising 30 mL of 0.5% bupivacaine, 0.3 mL of 0.1% adrenaline, and 90 mL of saline.

After surgery, patients were transferred to the post-anesthesia care unit. During the postoperative period, nonsteroidal anti-inflammatory drugs for analgesia were used in both groups of patients: acetaminophen 1 g 4 times a day and tramadol/dexketoprofen 75/25 mg 2 times a day. Opioids were administered to patients as supplementary analgesics when their subjective pain score, as measured by the visual analog scale, exceeded 5, following the exhaustion of other pain management strategies. Rescue opioids, such as pethidine or morphine, were given via intravenous intermittent infusion until satisfactory analgesic effects were attained or the maximum dosage was reached (pethidine 50 mg or morphine 10 mg).

### Assessment of outcomes

To reduce the impact of environmental factors on neuropsychological assessment results, all evaluations were conducted in a quiet setting. The assessment was carried out by a qualified outcomes evaluator in accordance with standardized protocols. The primary outcome was to compare cognitive function between the BIS and non-BIS groups at all assessment time points. Secondary outcomes included longitudinal changes in overall cognitive function and specific cognitive domains, as well as alterations in emotional status over time.

Neurocognitive performance was evaluated using the *Addenbrooke’s Cognitive Examination–Revised (ACE-R)* questionnaire, a detailed and validated battery that assesses multiple cognitive domains: attention and orientation (18 points), memory (26 points), verbal fluency (14 points), language (26 points), and visuospatial abilities (16 points). The maximum total score is 100 points. Higher scores indicate better cognitive performance. The minimal clinically important difference (MCID) for detecting POCD was defined as a 5-point decrease in ACE-R score. This detailed assessment was selected over broader screening instruments such as the Mini-Mental State Examination (MMSE) to enable a more precise analysis of specific cognitive impairments. MCID value was chosen according to the recommendations of the International Perioperative Cognition Nomenclature Working Group [5], POCD is defined as a decline of 1 standard deviation from baseline performance. Therefore, an MCID of 5 points was selected based on the Lithuanian adaptation and validation study of the ACE-R questionnaire [17] (age-adjusted mean score 91.59, SD 5.43). The mental function was assessed four times: preoperatively (PO) and at 2, 30, and 90 days after the knee arthroplasty (2, 30, and 90 POD).

The emotional status was evaluated preoperatively and at 30 POD. Anxiety and depressive symptoms were assessed using the Hospital Anxiety and Depression Scale (HADS), a validated self-report questionnaire designed to detect clinically relevant symptoms of anxiety and depression in hospital settings. The HADS consists of 14 items divided into two subscales: anxiety (HADS-A) and depression (HADS-D), each ranging from 0 to 21. Subscale scores were interpreted as follows: 0–7 indicated normal levels, 8–10 indicated borderline abnormal (possible anxiety or depression), and ≥ 11 indicated clinically significant (probable) anxiety or depression.

### Data collection and statistical analysis

The following demographic and perioperative characteristics of patients were collected: age, gender, education time, ASA status, comorbidities, propofol dose, duration of surgery, and BIS value. Moreover, the neuropsychological test results were recorded.

Sample size was calculated a priori using G*Power version 3.1.9.7. With a statistical power (1–β error) of 0.80, a significance level (α) of 0.05, and an effect size of 0.69, the minimum required sample size was 36 participants per group. The effect size (*d* = 0.69) was calculated by dividing the expected clinically meaningful difference in ACE-R scores (5 points) by the pooled standard deviation (7.21 points) obtained from the Lithuanian adaptation and validation study of the ACE-R questionnaire results [17] using the equation:

$$\mathrm{S}\mathrm{D}\mathrm{p}\mathrm{o}\mathrm{o}\mathrm{l}\mathrm{e}\mathrm{d}=\sqrt{\frac{\left({n}_{1}-1\right)S{D}_{1}^{2}+\left({n}_{2}-1\right)S{D}_{2}^{2}}{{n}_{1}+{n}_{2}-2}}$$

where

n_1_, SD_1_—score mean in the general population is 91.04 (SD 7.17), *n* = 315;

n_2_, SD_2_—score mean in group with mild cognitive dysfunction is 74.06 (8.09), *n* = 16.

A total of 42 participants per group is required to account for a possible 15% dropout rate.

Statistical analysis was performed using *IBM SPSS V.30.0 (IBM Corp., Armonk, NY, USA)*. Baseline demographic and clinical characteristics were summarized, and the balance between BIS and non-BIS groups was assessed using absolute standardized differences. An absolute standardized difference of less than 0.10 was considered indicative of negligible imbalance between groups. Categorical variables were compared between two dependent groups using the McNemar-Bowker test. The Shapiro–Wilk test was used to assess the normality of continuous data. The Mann–Whitney* U* test was applied to compare non-normally distributed data between two independent groups. The Friedman test was applied to compare data across four time points, with a Bonferroni correction for pairwise comparisons. The Spearman’s rank correlation coefficient was used to assess the association between non-normally distributed variables. Categorical variables are presented as frequency (*n*) and percentages (%), normally distributed data as mean and standard deviation (SD), and non-normally distributed data as median and minimum and maximum values (min.-max.). Also, the Kendall’s* W* effect size for the Friedman test, and Cramer’s* V* for the proportion were reported. The effect sizes were reported together with 95% confidence intervals (CI). A *p*-value of less than 0.05 was regarded as indicative of statistical significance.

## Results

**Fig. 1:**
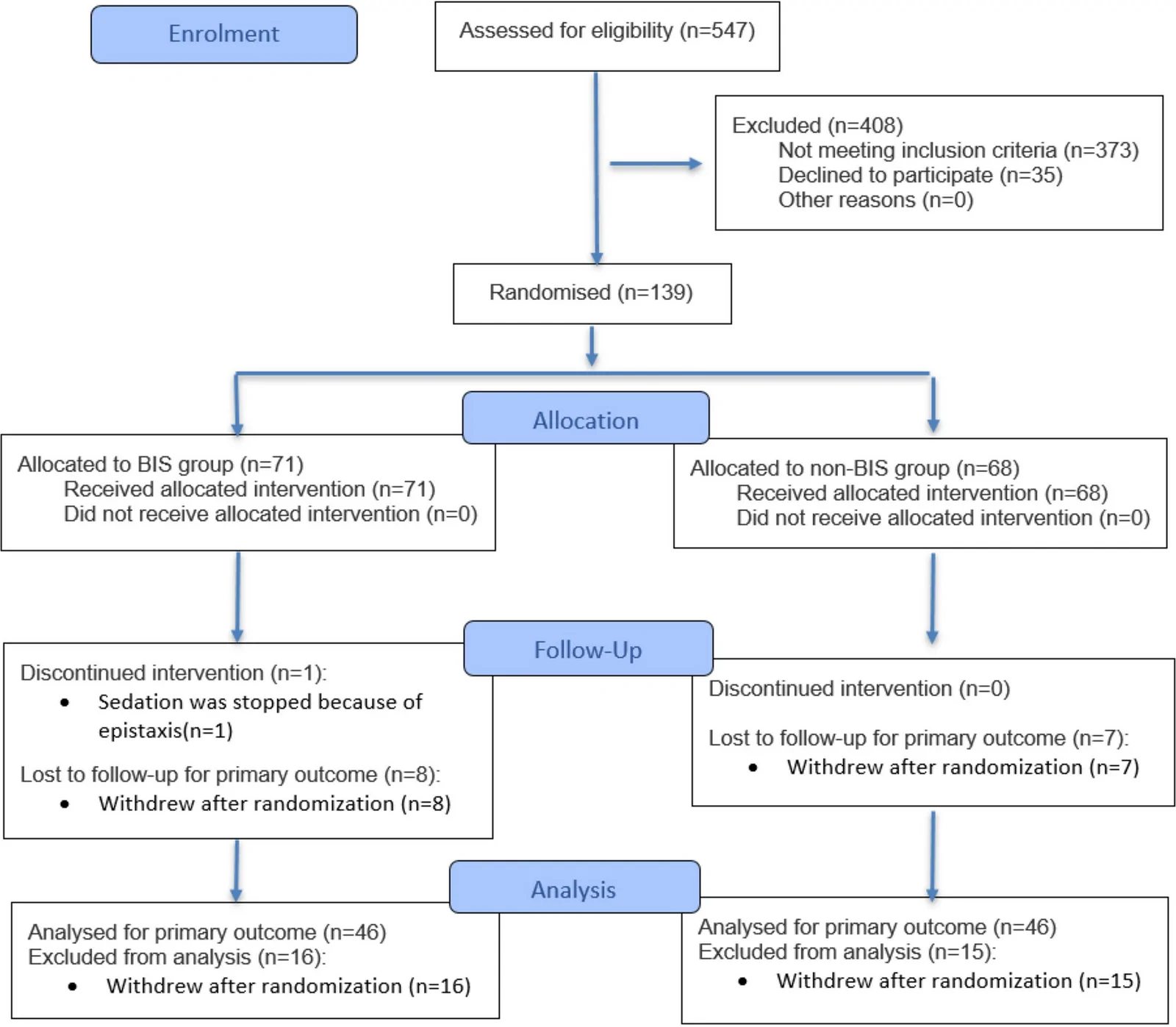
CONSORT flow diagram of the study

Patients were enrolled between February 2024 and May 2025. During the study period, a total of 547 patients were screened for eligibility. Of these, 373 were excluded for not meeting the inclusion criteria: 223 were excluded due to cognitive health disorders and/or the use of benzodiazepines or other psychotropic medications, and 150 were excluded because they were younger than 65 years. Among the remaining patients, 35 declined to participate in the study before randomization. Consequently, 139 patients were randomized into two groups: 71 to the BIS group and 68 to the non-BIS group. During follow-up, attrition occurred in both groups. Ultimately, 46 patients in each group completed the study and were included in the final analysis for the primary outcome. The CONSORT flow diagram detailing participant enrollment, allocation, follow-up, and analysis is shown in Fig. 1.

Baseline demographic and clinical characteristics are presented in Table 1. According to the absolute standardized differences, the groups were substantially imbalanced with respect to sedation depth (BIS value), moderately imbalanced in age, gender, years of education, and duration of surgery, and only slightly imbalanced in several comorbidities and ASA physical status.

**Table 1:** Comparison of baseline characteristics between the two groups

	BIS group *n* = 46 (50%)	Non-BIS group *n* = 46 (50%)	Absolute standardized difference
Age, years	71 (6)	73 (5)	0.36
Gender, female	35 (76.1)	41 (89.1)	0.35
Education, years,	12 (2.4)	11.4 (3.3)	0.2
ASA physical status,			
I	6 (13)	5 (10.9)	0.06
II	36 (78.3)	39 (84.8)	0.17
III	4 (8.7)	2 (4.3)	0.18
Comorbidities			
Hypertension	38 (82.6)	40 (87)	0.12
Ischemic heart disease	10 (21.7)	9 (19.6)	0.05
MI	4 (8.7)	2 (4.3)	0.18
Diabetes mellitus	9 (19.6)	10 (21.7)	0.05
Stroke	3 (6.5)	3 (6.5)	0.000
Propofol, mg	293 (51.9)	297 (60.2)	0.07
Duration of surgery, min	143 (14)	140 (12.5)	0.23
BIS value	79.3 (2.2)	66 (5.4)	3.29
Rescue opioids	3 (6,5)	6 (13)	0.21

The data were presented as *n* (%) and mean (SD)

*Abbreviations*:* ASA* American society of anesthesiologists, *MI* myocardial infarction, *BIS* bispectral index, *SD* standard deviation

### Primary outcome

The median of the total ACE-R score in the BIS versus non-BIS group was 87 (65–96) versus 87 (48–96) preoperatively, 89 (64–99) versus 88 (45–98) on POD 2, 91 (62–98) versus 90.5 (40–98) on POD 30, and 92 (70–98) versus 91 (40–99) on POD 90. No statistically significant differences between the groups were observed at any assessment time point (PO: *p* = 0.187; POD 2: *p* = 0.439; POD 30: *p* = 0.931; POD 90: *p* = 0.485).

### Secondary outcomes

**Fig. 2:**
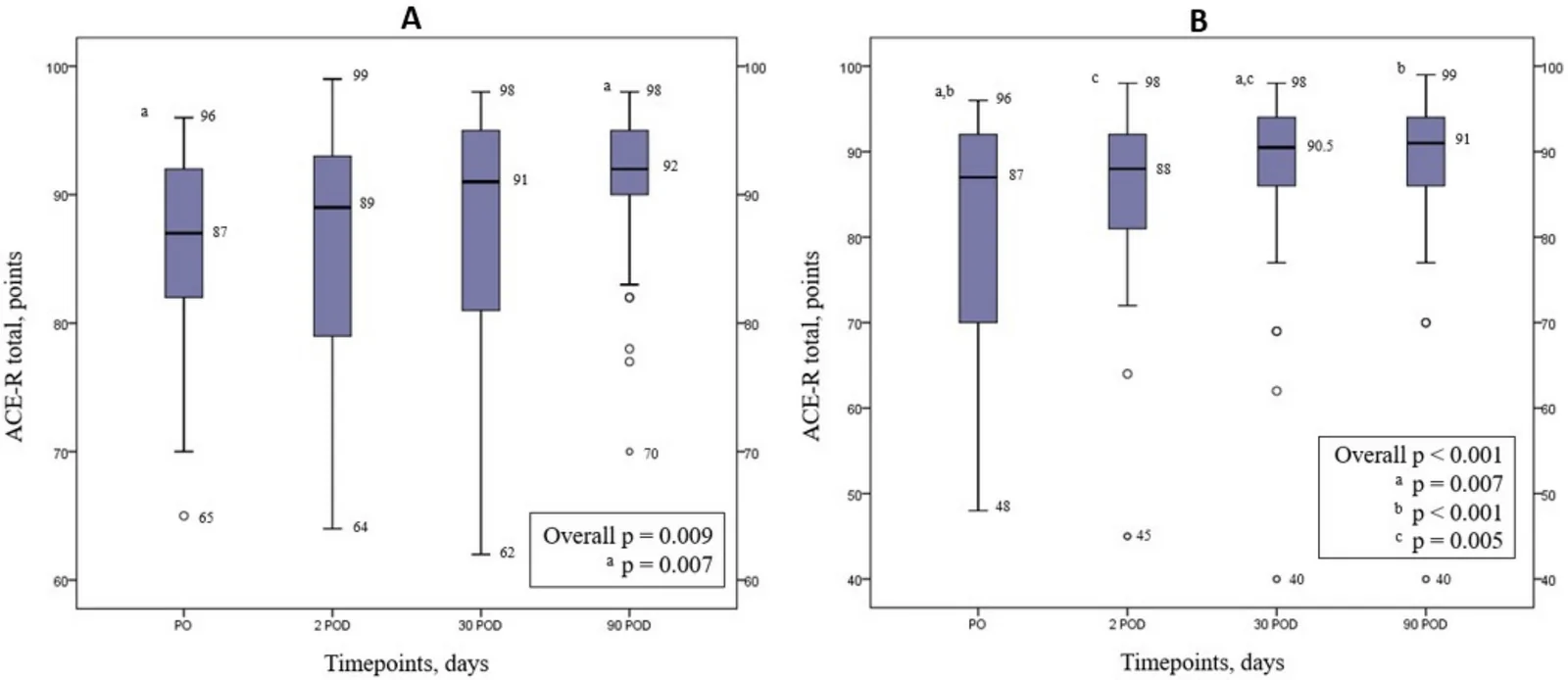
Longitudinal comparison of total ACE-R scores between different time points within each study group. **A** In the BIS group. **B** In the non-BIS group. Overall differences within each group were assessed using the Friedman test; ^a,b,c^ Friedman test with Bonferroni correction for pairwise comparisons. PO (preoperatively); POD (postoperative day)

Within the BIS group (Fig. 2A), total ACE-R scores were statistically significantly different between the two time points (*p* = 0.009; Kendall’s* W* effect size = 0.526, 95% CI [0.381; 0.652]): higher at 90 POD than at PO (*p* = 0.007). In the non-BIS group (Fig. 2B), the total ACE-R scores demonstrated significant increases at several time points (*p* = < 0.001; Kendall’s* W* effect size = 0.183, 95% CI [0.102; 0.296]): from PO to 30 POD (*p* = 0.007), from PO to 90 POD (*p* < 0.001), and from 2 to 30 POD (*p* = 0.005).

A detailed summary of the comparison of all cognitive domains evaluated (attention and orientation, memory, language, verbal fluency, and visuospatial skills) is presented in Table 2.

**Table 2:** Comparison of all cognitive domains between all timepoints in BIS and non-BIS groups

	PO	2 POD	30 POD	90 POD	*p* values	^‡^ Effect size [95% CI]
Attention and orientation						
BIS	18 (15–18)	18 (14–18)	18 (13–18)	18 (16–18)	^†^ 0.001 ^a^ > 0.05	0.125 [0.058; 0.214]
Non-BIS	18 (9–18)	17 (7–18)	18 (10–18)	18 (10–18)	^†^ 0.001 ^a^ > 0.05	0.112 [0.052; 0.204]
Memory						
BIS	18.5 (8–24) ^a,b^	21 (12–26) ^a,c^	22 (10–26)	22 (12–26)^b,c^	^†^ < 0.001 ^a^ 0.002 ^b^ < 0.001 ^c^ 0.022	0.227 [0.135; 0.361]
Non-BIS	18 (4–24) ^a,b^	20 (9–26) ^c^	22 (4–26) ^b^	22 (4–26) ^a^	^†^ < 0.001 ^a^ < 0.001 ^b^ < 0.001 ^c^ 0.036	0.233 [0.142; 0.370]
Language						
BIS	24 (17–26)	25 (19–26)	24 (20–26)	25 (20–26)	^†^ 0.027 ^a^ > 0.05	0.067 [0.021; 0.148]
Non-BIS	24 (14–26)	24 (13–26)	24 (14–26)	24 (13–26)	^†^ 0.128	0.08 [0.028; 0.163]
Verbal fluency						
BIS	11 (5–14)	11.5 (6–14)	11 (4–14)	12 (7–14)	^†^ 0.258	0.029 [0.006; 0.084]
Non-BIS	11 (5–20) ^a^	11 (6–14)	11.5 (3–14)	12 (3–14) ^a^	^†^ 0.012 ^a^ 0.032	0.041 [0.011;0.105]
Visuospatial skills						
BIS	16 (14–16) ^a^	15 (9–16)	15 (11–16) ^a^	15 (12–16)	^†^ < 0.001 ^a^ 0.004	0.131 [0.064; 0.225]
Non-BIS	15 (8–16)	14 (9–16)	15 (9–16)	15 (10–16)	^†^ 0.091	0.047 [0.014; 0.116]

The data are presented as median (minimum–maximum values).

*Abbreviations*:* PO* preoperatively, *POD* postoperative day

^†^Friedman test

^a,b,c^ Friedman test with *Bonferroni* correction for pairwise comparison

^‡^Kendall’s* W* effect sizes for the Friedman test with 95% confidence intervals

Memory scores changed significantly over time in both groups. In the BIS group, there were statistically significant increases in median memory score between PO and 2 POD (*p* = 0.002), PO and 90 POD (*p* < 0.001), and between 2 and 90 POD (*p* = 0.022). Similarly, in the non-BIS group, significant increases in median memory scores were identified between PO and 2 POD (*p* < 0.001), PO and 30 POD (*p* < 0.001), and between 2 and 30 POD (*p* = 0.036).

In the BIS group, verbal fluency scores were stable throughout the study period, with no significant time-related changes (*p* > 0.05). In contrast, the non-BIS group showed a modest but statistically significant difference between PO and 90 POD assessments (*p* = 0.032). However, the other comparisons were not statistically significant.

In the BIS group, visuospatial skills changed over time, with a statistically significant difference between the PO and 30 POD assessments (*p* = 0.004). In the non-BIS group, visuospatial scores remained relatively stable across all time points, with no statistically significant differences detected (*p* > 0.05).

Scores for attention and orientation, and language remained stable over time in both groups. No statistically significant changes were observed across the four assessment points in either the BIS or non-BIS group (*p* > 0.05).

**Fig. 3:**
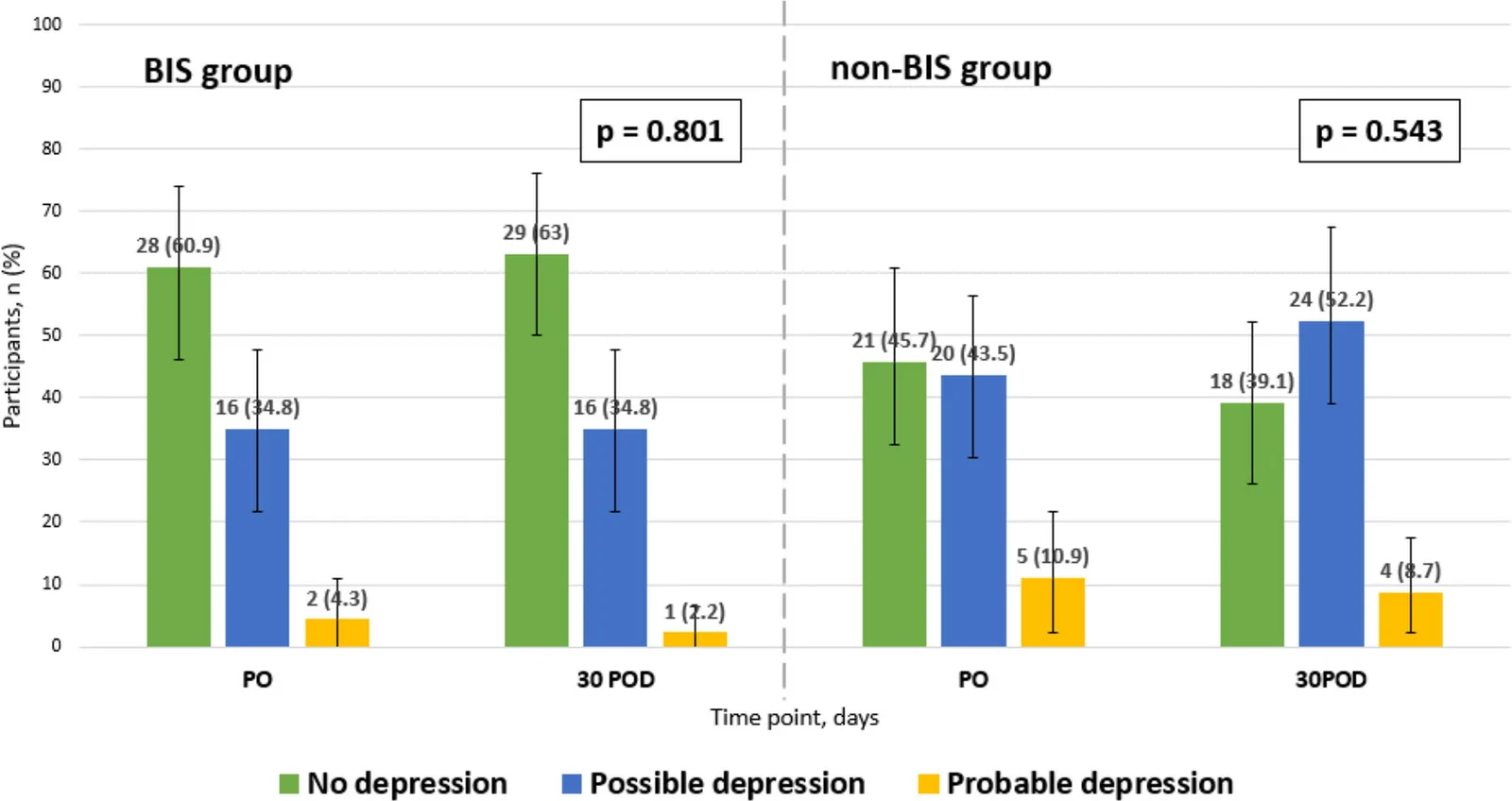
Comparison of the HADS-A score between two different time points in the BIS and non-BIS groups. The data are presented as *n* (%) and compared by the McNemar-Bowker. PO (preoperatively); POD (postoperative day)

**Fig. 4:**
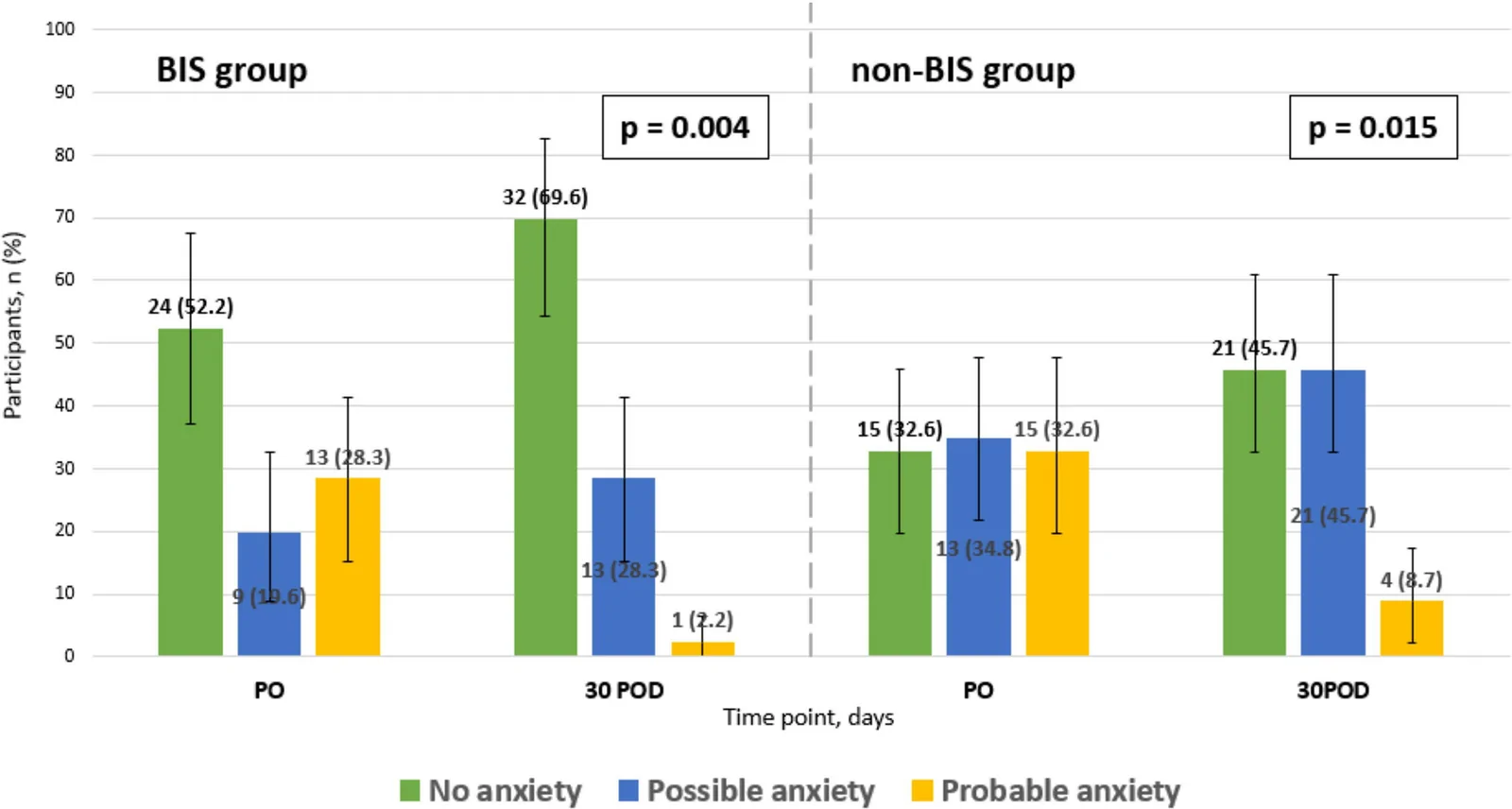
Comparison of the HADS-D score between two different time points in the BIS and non-BIS groups. The data are presented as *n* (%) and compared by the McNemar-Bowker test. PO (preoperatively); POD (postoperative day)

In the BIS group (Fig. 3), the proportion of patients without anxiety increased statistically significantly from 52.2% preoperatively to 69.6% at 30 POD, whereas the proportion with probable anxiety decreased considerably from 28.3 to 2.2% (*p* = 0.004; Cramer’s *V* 0.385, 95% CI [0.238; 0.721]). In the non-BIS group (Fig. 3), no anxiety increased statistically significantly from 32.6 to 45.7%, and probable anxiety decreased from 32.6 to 8.7% between PO and 30 POD (*p* = 0.015; Cramer’s *V* 0.263, 95% CI [0.166; 0.467]). There were no statistically significant differences in the distribution of depression categories between PO and 30 POD in either group (BIS group: *p* = 0.801; Cramer’s *V* 0.426, 95% CI [0.3; 0.786], non-BIS group: p = 0.543; Cramer’s *V* 0.501, 95% CI [0.274; 0.739]). Detailed results are presented in Fig. 4.

Furthermore, relationships between the total ACE-R score and anxiety were detected preoperatively and on 30 POD. In both time points, we detected a weak negative correlation PO (*r* = − 0.336, *p* < 0.001, 95% CI [− 0.507; − 0.126]) and on 30 POD (*r* = − 0.237, *p* < 0.023, 95% CI [− 0.445; − 0.031]).

## Discussion

In this study, the effects of two strategies of monitoring sedation depth—BIS-guided sedation and clinical assessment-based sedation—were compared. No deterioration was observed in either overall cognitive function or specific cognitive domains. Nevertheless, the study identified non-specific changes in cognitive performance, which were likely attributable to psychological factors and the effects of repeated testing.

Firstly, the observed deterioration in preoperative cognitive performance and subsequent changes may be associated with perioperative anxiety. Anxiety prior to surgery is common among patients undergoing TKA and may adversely influence outcomes on cognitive assessments. Although only a weak correlation between anxiety levels and the total ACE-R score was identified in both groups, an analysis of absolute values demonstrated a significant decline in the number of patients experiencing anxiety at 30 POD in both the BIS (probable anxiety from 28.3 to 2.2%) and non-BIS groups (probable anxiety from 32.6 to 8.7%). This reduction in anxiety may have contributed to improved postoperative test performance.

Secondly, the findings may be influenced by test-related factors. The Addenbrooke’s Cognitive Examination–Revised (ACE-R) is a validated and extensively employed instrument for detecting subtle postoperative cognitive changes and has been translated and validated into Lithuanian. However, like most other questionnaires, the ACE-R has a notable limitation: the absence of multiple equivalent versions. Repeated administration of the same test over relatively brief intervals may lead to a learning or practice effect, whereby participants recall prior responses and perform better on subsequent assessments. Consequently, improved postoperative outcomes may reflect increased familiarity with the test. Furthermore, if participants indeed remembered test items, this indicates that sedation did not adversely affect memory function, a fundamental domain frequently associated with POCD.

Furthermore, in this study, the observation that sedation depth was deeper in the non-BIS group is secondary (collateral). In the context of a negative study (i.e., no cognitive decline was detected in either group), this finding indicates that even deeper sedation does not impair cognitive function after elective knee arthroplasty.

In summary, these findings suggest that clinically guided sedation does not associate with an increased risk of postoperative cognitive dysfunction (POCD) when compared to BIS-guided sedation. This implies that using BIS monitoring to titrate sedation levels does not improve postoperative or post-procedural cognitive outcomes relative to conventional assessment methods based on patient responses and validated sedation scales. Both methodologies yield comparable outcomes concerning the preservation of cognitive function, safety, and recovery profiles.

To our knowledge, this is the first study to compare the effects of two sedation depth monitoring strategies (BIS-guided sedation and clinical assessment based sedation) on cognitive function in patients undergoing elective total knee replacement surgery under spinal anesthesia. Previous studies involving orthopedic patients have differed in several important aspects. Some have focused on the effects of the depth of general anesthesia (deep versus light sedation) on cognitive outcomes [13, 18], while others have examined patients undergoing emergency orthopedic surgical procedures [14, 19, 20]. In addition, other studies have investigated different sedative agents [14, 21], and the methods used to assess cognitive function have also varied significantly. Therefore, these studies are not directly comparable to our work due to differences in patient populations, surgical urgency, methodological approaches, and outcome measures.

This study is subject to several limitations. Firstly, the relatively small sample size may have constrained statistical power, and data were collected from a single location, which limits the generalizability of the findings. Secondly, certain factors, such as participants’ anxiety levels, could not be controlled and may have influenced the results. Moreover, during the study, there is no way to avoid training. While the questionnaire employed was appropriate, alternative versions of the instrument exist that could provide different perspectives. Despite these limitations, the findings offer valuable preliminary evidence and can guide future research with larger, more diverse populations and more rigorously controlled conditions.

## Conclusion

This study did not demonstrate superiority of BIS-controlled sedation over clinically guided sedation with respect to cognitive outcomes in elderly patients undergoing elective knee arthroplasty under spinal anesthesia. Further randomized controlled studies are needed to confirm these findings.

## Data Availability

All data generated or analysed during this study are included in this published article.

## Abbreviations

POCD: Postoperative cognitive dysfunction
TKA: Total knee arthroplasty
SA: Spinal anesthesia
BIS: Bispectral index
ACE-R: Addenbrooke’s Cognitive Examination–Revised
PO: Preoperatively
POD: Postoperative day
HADS: Hospital Anxiety and Depression Scale
